# An Acute Discovery of Fourth Ventricle Outflow Obstruction in Previously Idiopathic Chronic Hydrocephalus: A Case Report

**DOI:** 10.7759/cureus.73557

**Published:** 2024-11-12

**Authors:** João Pedro Oliveira, João Ramos, Mário Campos, Pedro Calvão-Pires, Pedro Cunha

**Affiliations:** 1 Neurosurgery, Centro Hospitalar de Lisboa Ocidental, Lisbon, PRT; 2 Neuroradiology, Centro Hospitalar de Lisboa Ocidental, Lisboa, PRT; 3 Neurosurgery, Unidade Local de Saúde Lisboa Ocidental - Hospital Egas Moniz, Lisboa, PRT; 4 Neuroradiology, Centro Hospitalar de Lisboa Ocidental, Lisbon, PRT

**Keywords:** adult hydrocephalus, foramen of magendie, hydrocephalus, management of obstructive hydrocephalus, neuro-imaging

## Abstract

Hydrocephalus is the disruption of cerebral spinal fluid homeostasis, representing a common neurosurgical illness. Up to 10% have no identifiable cause, with fourth ventricle outflow obstruction (FVOO) being an extremely rare subtype. A 31-year-old male with a history of idiopathic hydrocephalus for over 10 years with the need for a ventriculoperitoneal shunt had shown progressively enlarged tetraventricular ventriculomegaly. There was no history of meningitis, traumatic injuries, or surgeries besides the shunt placement. Given clinical worsening with diplopia and gait impairment and imaging suggestive of shunt failure, the patient underwent surgery for a replacement. In the following days, the patient worsened and became lethargic. Bilateral frontal external ventricular drainages were placed for acute hydrocephalus due to tetraventricular haemorrhage, although ventricular enlargement kept progressing. Brain magnetic resonance imaging (MRI) showed a membrane in the Magendie foramen region with an apparently unobstructed aqueduct and no transependymal edema. The surgical team opted for a suboccipital craniectomy for membrane fenestration. Post-operative computed tomography (CT) scans showed a stark reduction of the ventricles. Within one week, the patient had an almost complete recovery and was discharged. One year later, the patient is asymptomatic with no need for acetazolamide or shunt. Hydrocephalus is a rather common neurosurgical pathology presenting serious comorbidity. It is amenable to very effective and potentially curative treatment, and therefore efforts should be made to find its cause. Here, we reported a rather challenging referral of an acute decompensation of a chronic hydrocephalus with no previous cause identified. Ultimately, in the midst of this chronically altered cerebrospinal fluid (CSF) dynamics, the use of advanced high-resolution imaging proved crucial and allowed adequate treatment for the true cause of the hydrocephalus. We hope to raise awareness of the possible existence of membranes in the foramen of Magendie and its unusual presentation.

## Introduction

Hydrocephalus concerns the disruption of either production, communication, or reabsorption of cerebrospinal fluid (CSF) usually resulting in the abnormal accumulation and enlargement of the cerebral ventricles. In the realm of neurosurgery, hydrocephalus is rather frequent with an incidence of 1:1000 in children and up to 6% of the elderly. It is also rather costly for healthcare systems, at an estimated yearly expense of US$2 billion [1,2].

Hydrocephalus can be classified as non-communicating or obstructive and communicating or non-obstructive, as well as according to etiology. The most common causes are hemorrhage, congenital aqueduct stenosis, and brain tumors. However, a large portion remains unclassified or idiopathic, representing almost 10% of cases. Fourth ventricle outflow obstruction (FVOO) is an extremely rare condition of non-communicating hydrocephalus, presenting with an obstructive membrane overlying the foramen of Magendie or Luschka, although perhaps of increasing incidence with the advent of high-resolution volumetric imaging, which allows a more detailed inspection of the Magendie and Luschka foramina [3]. We present a case of a 31-year-old male with an adolescent-onset hitherto idiopathic hydrocephalus recently diagnosed with idiopathic FVOO.

## Case presentation

A 31-year-old male with a history of idiopathic hydrocephalus for over 10 years with a ventriculoperitoneal shunt (VPS) presented as an outpatient with increasing ventriculomegaly detected by recent CT (not shown) and diplopia and gait impairment, suggestive of shunt failure. The remainder clinical history was unremarkable.

**Figure 1:**
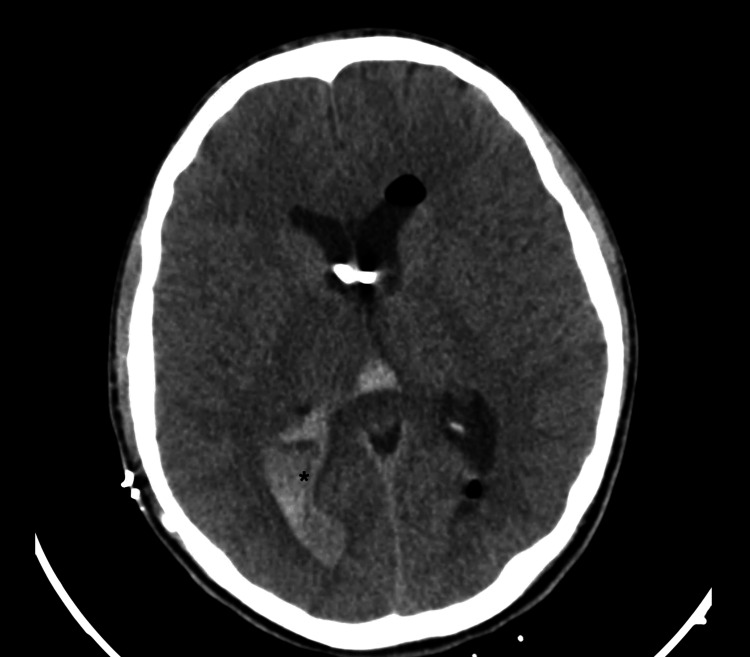
Post-surgical CT scan showing significant intraventricular hemorrhage (*).

The patient was admitted for a shunt replacement. In the first 24 hours after surgery, there was acute hydrocephalus due to intraventricular hemorrhage (Figure 1) with clinical worsening (Glasgow Coma Scale (GCS) of 4). The patient greatly improved over a few days (GCS 13) after the placement of bilateral frontal external ventricular drains (EVDs).

**Figure 2:**
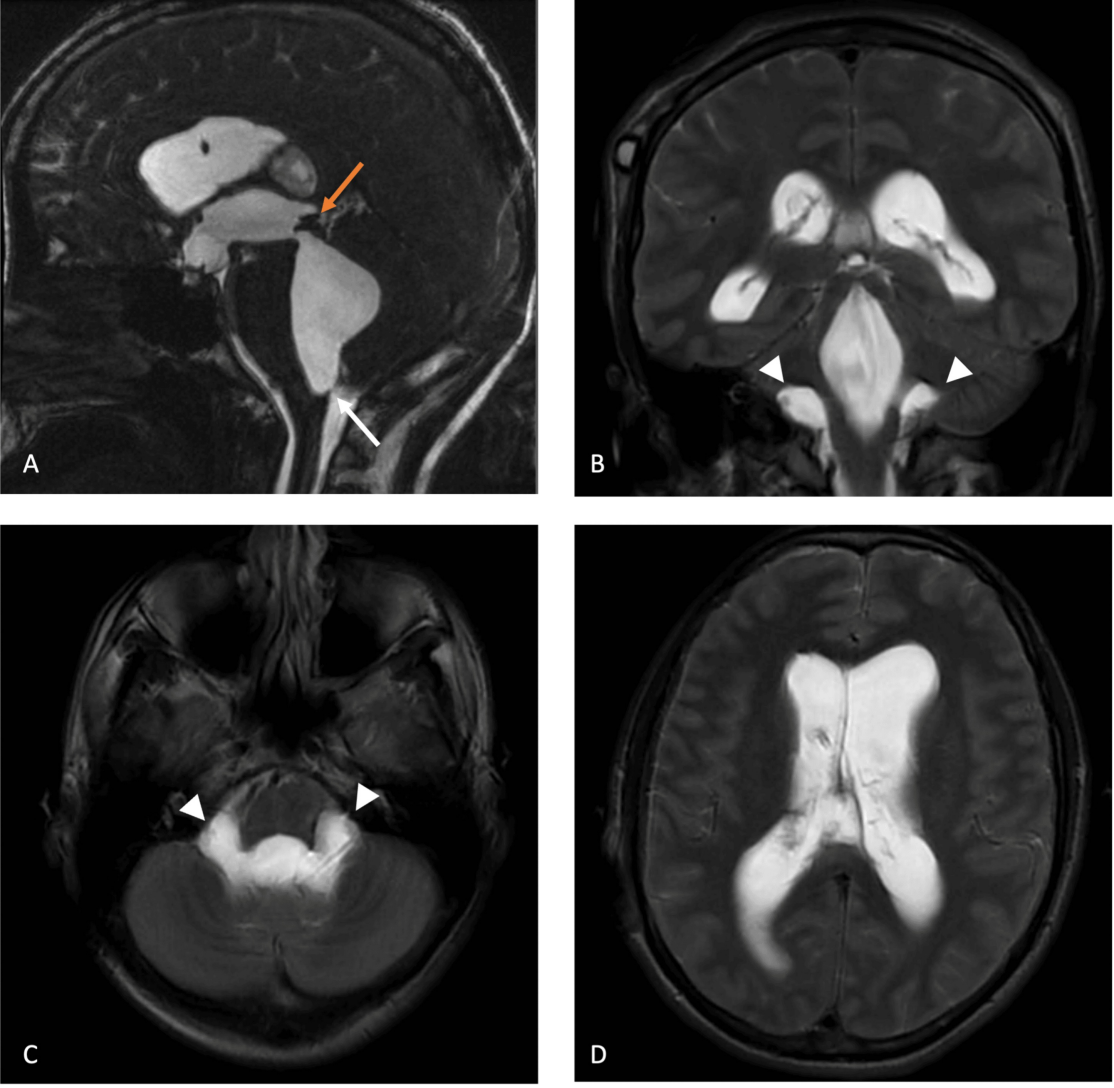
Brain MR showing a linear structure at the region of the foramen of Magendie, highly suggestive of a membrane. A: a dilated fourth ventricle with a membrane at the foramen of Magendie (white arrow) and patent Sylvian aqueduct (orange arrow). B, C: dilated Luschka foramina (arrowheads). D: no transependymal edema.

**Figure 3:**
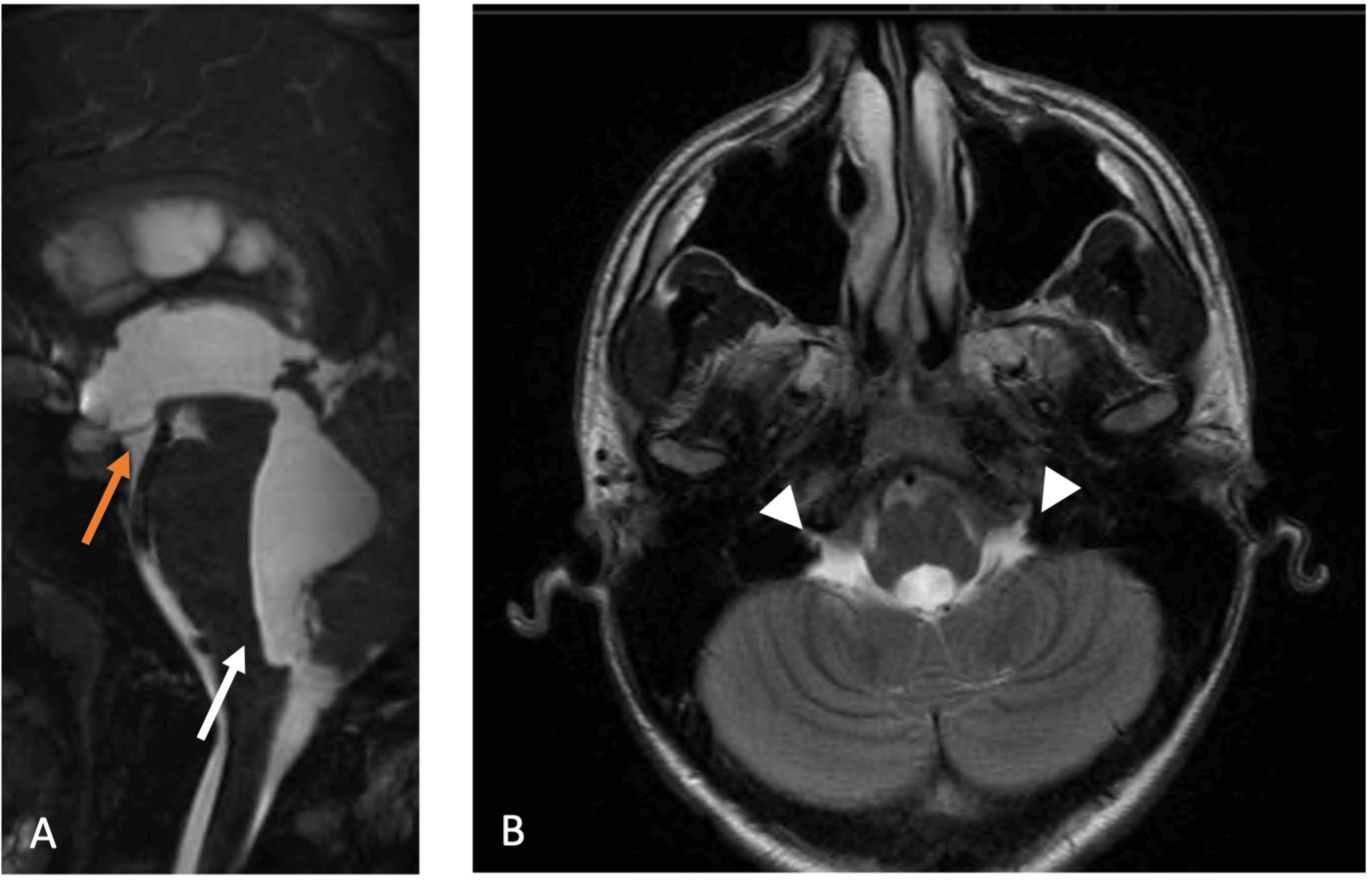
MRI from 2011 with T2-weighted submillimetre volumetric high-resolution imaging showing no clear membrane. A: bowing of the medulla (white arrow) and a low position of the floor of the third ventricle (orange arrow). B: Luschka foramina slightly enlarged (arrowheads).

Serial CT imaging showed progressively enlarging tetraventricular hydrocephalus, with a maximum Evans index of 0.37 (vs. 0.30 on the latest pre-operative imaging). A brain MR (Figure 2) confirmed the ventriculomegaly and excluded aqueduct stenosis. A 3D fast imaging employing steady-state acquisition (FIESTA) demonstrated a linear structure at the region of the foramen of Magendie (Figure 2A, white arrow), highly suggestive of a membrane. In addition, the Luschka foramina showed progressive enlargement compared to previous CT and MR, possibly suggesting diverticular dilatation, although no additional membranes were visualized. Surprisingly, there was no transependymal edema (Figure 2D). Upon revisiting the patient’s health records, a 3D FIESTA from 2011 was available (Figure 3), which demonstrated an anterior bowing of the medulla, suggesting tethering, although there was no clear membrane.

**Figure 4:**
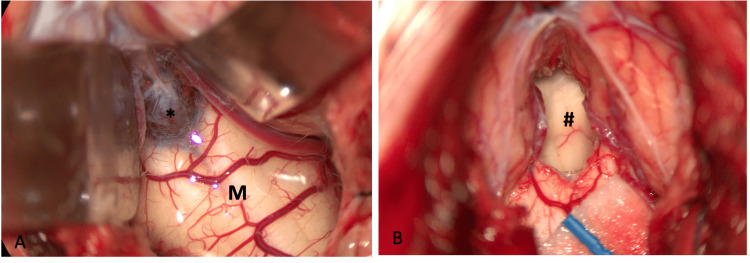
Intra-operatory findings Intra-operatory photographs demonstrating a membrane at the foramen of Magendie (A, *), which was fenestrated and patent at the end of the surgery with the fourth ventricle now visible (B, #). M – medulla oblongata

A midline incision and suboccipital craniectomy were performed to expose the floor of the fourth ventricle. On opening the dura, the cisterna magna, and separating the tonsils, a thickened dura and arachnoid were apparent. Upon visualization of the Magendie foramen region, a thick membrane (Figure 4A) with rich vascularization completely obstructing the foramen was identified under the cerebellar tonsils. This membrane was fenestrated successfully, following which the floor of the fourth ventricle and a patent Magendie foramen could be seen at the end (Figure 4B).

**Figure 5:**
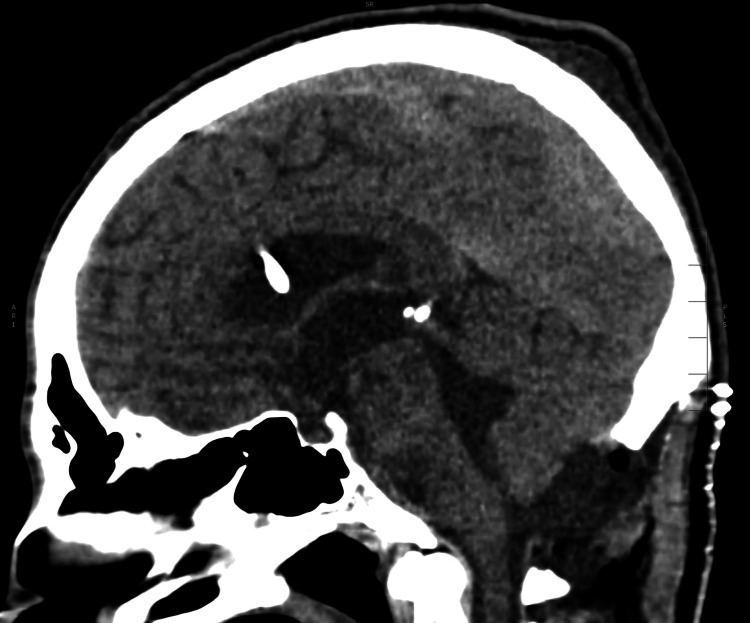
CT scan one week after surgery showing stark ventricle reduction. Fourth ventricle (#) now of normal size, with repositioning of the floor of the third ventricle (orange arrow). There was also reduction of the bowing of the medulla (white arrow). Usual post-surgical changes are also seen.

A week later, a CT showed stark ventricle reduction (Figure 5) and was accompanied of marked clinical improvement. Both EVDs were clamped and ultimately removed, and one week after surgery, the patient was discharged with an almost full recovery, presenting just mild diplopia.

**Figure 6:**
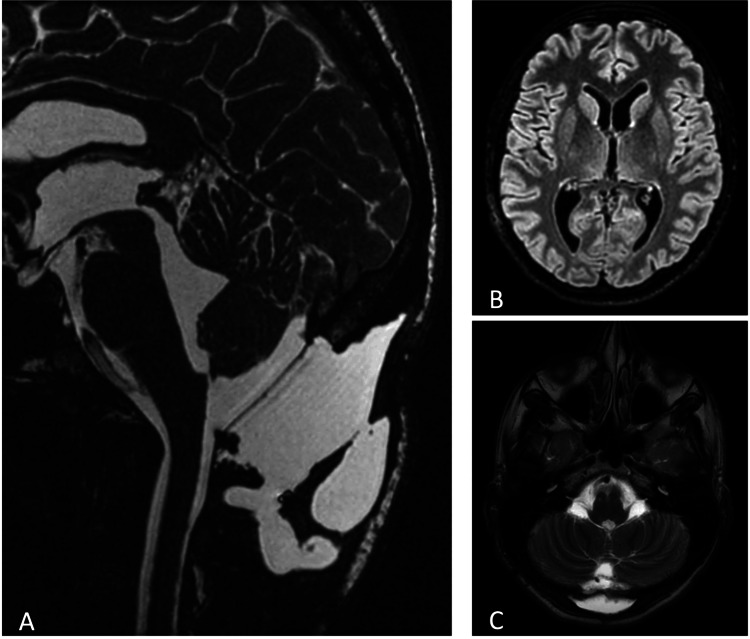
MRI at 1.5 months after membrane fenestration with no obstruction of the foramen of Magendie or signs of hydrocephalus. A: Both the fourth ventricle and floor of the third ventricle look normal. B: Supratentorial ventricles are normal, with no transependymal oedema. C: Some dilatation of the Luschka foramina is still observed. A slight enlargement of the pseudomeningocele is noted.

About one month later, the patient presented in the emergency room with a new onset of headache and nausea with increased intracranial pressure (25 cm H_^2^_O on lumbar puncture in the lateral recumbent position), which prompted treatment with acetazolamide. A new MRI (Figure 6) confirmed no obstruction of the foramen of Magendie, stable cerebral ventricle size, and no transependymal edema or new lesions. In a few days, the patient was much improved and intracranial pressure had fallen to 19 cm H_2_O. He was then discharged and until the time of writing (over six months) has not shown clinical deterioration.

## Discussion

FVOO is a rather rare form of non-communicating hydrocephalus first thought to be reported by Dandy in 1921 [4], presenting with a membrane at the foramen of Magendie. This can sometimes be accompanied by further membranes at the foramina of Luschka. Of note, this pathology is rather different from the trapped fourth ventricle [5], where the aqueduct is also obliterated, the latter leaving the fourth ventricle isolated from the remainder of the CSF pathway.

Given the advent of high-resolution submillimetre volumetric imaging such as with 3D FIESTA and similar sequences (e.g., CISS, SPACE), there has been an increase in the diagnosis of abnormal membranous structures along CSF pathways, namely, in the Magendie or Luschka foramina. In fact, Dinçer et al. [3] reclassified previously communicating hydrocephalus to non-communicating in about 60% of the cases with the aid of 3D CISS as opposed to just using conventional imaging. As for our case, it was not until we reviewed this sequence that we were able to find the membrane. This makes the case for the crucial role high-resolution imaging in the modern day has in finding a possible cause amenable to potentially curative treatment.

Clinically speaking, FVOO has quite a variable onset ranging from childhood [6-10] up to middle age [11], with presentation ranging from acute [12] to more delayed [13]. Some patients have a history of previous central nervous system infections - with a recent case associated with recent prior COVID-19 infection [14], or intracranial hemorrhages, which are known to create adhesions, while others have no identifiable causes such as in this patient. For the most part, the idiopathic cases are believed to be congenital, raising the possibility of atresia of the fourth ventricle foramina [15]. However, occasionally, there is imaging prior to the FVOO-related clinical presentation with demonstrable normal communication between the subarachnoid space and the fourth ventricle, which provides evidence for an acquired etiology in some of the cases [10].

Most of the symptoms are due to compression of the brainstem or the cerebellum by the enlarged fourth ventricle, usually with cranial nerve palsies or gait impairment along with signs of high intracranial pressure. Very rarely, there have also been cases of cervical spinal cord edema, presumably due to further CSF flow disruption within and around the central canal in the upper spinal cord [13,16].

The course our patient went through is rather interesting and unlike what has been described in the literature. Previous to the referral, the hydrocephalus was considered idiopathic and treated with a VPS - which is one of the recommended treatments for FVOO [17,18] - with clinical improvement. Even after reviewing the images 10 years ago, a membrane at the foramen of Magendie is not immediately apparent, which does not entirely exclude its existence, however, as the indirect signs are telling.

After 10 years, our patient showed worsening hydrocephalus suggestive of shunt failure, which was promptly replaced. As EVDs were not effective in alleviating refractory hydrocephaly in the immediate post-op period probably due to acute (intraventricular blood) on chronic (FVOO) hydrocephalus causes, further investigation was performed. Throughout the literature, there has been controversial discussion regarding treatment options. These include more physiological approaches such as membrane fenestration which re-establishes the normal anatomy of the CSF pathway [8,12,19], and other surgical alternatives such as VPS or endoscopic third ventricle ventriculostomy (ETV) [6,10], the latter being the alternative with the lowest recurrence rate [17]. There are always considerations to be made for the type of surgery regarding the patient’s condition and anatomy. Specifically, an ETV was deferred in our patient since the premammillary and prepontine spaces were severely diminished due to the mass effect exerted by the extremely enlarged fourth ventricle. This significantly increases the risk of complications in ETV, especially given the proximity of the basilar and posterior cerebral arteries. Therefore, a suboccipital craniotomy was performed with successful fenestration of the membrane at the Magendie foramen.

Our case provides evidence of the importance of long-term follow-up of these cases. To our knowledge, there are very few cases in the literature with symptomatic recurrence [20] (mostly within six to 24 months) although these have overall short follow-up periods reported. However, it is humbling to think recurrences happen and that these may occur decades after the initial presentation for numerous reasons. In addition to the baseline condition, multiple neurosurgical approaches along the way may also severely disturb normal CSF dynamics and its ability to be adequately restored, making it all the more important for these cases to be reported and shared among the scientific community.

## Conclusions

In summary, our goal was to raise awareness of FVOO in the context of high-resolution imaging and to motivate the community to actively seek treatable causes of hydrocephalus. We presented a long-term follow-up of a case that provides compelling evidence that membrane fenestration is a safe and effective treatment for this condition, even following previous surgeries that may have disrupted CSF dynamics. Furthermore, given the limited understanding of FVOO, we advocate for routine surveillance in this setting to mitigate potential complications.
